# Concordant Association of Insulin Degrading Enzyme Gene (*IDE*) Variants with *IDE* mRNA, Aß, and Alzheimer's Disease

**DOI:** 10.1371/journal.pone.0008764

**Published:** 2010-01-19

**Authors:** Minerva M. Carrasquillo, Olivia Belbin, Fanggeng Zou, Mariet Allen, Nilufer Ertekin-Taner, Morad Ansari, Samantha L. Wilcox, Mariah R. Kashino, Li Ma, Linda H. Younkin, Samuel G. Younkin, Curtis S. Younkin, Toros A. Dincman, Melissa E. Howard, Chanley C. Howell, Chloe M. Stanton, Christopher M. Watson, Michael Crump, Veronique Vitart, Caroline Hayward, Nicholas D. Hastie, Igor Rudan, Harry Campbell, Ozren Polasek, Kristelle Brown, Peter Passmore, David Craig, Bernadette McGuinness, Stephen Todd, Patrick G. Kehoe, David M. Mann, A. David Smith, Helen Beaumont, Donald Warden, Clive Holmes, Reinhard Heun, Heike Kölsch, Noor Kalsheker, V. Shane Pankratz, Dennis W. Dickson, Neill R. Graff-Radford, Ronald C. Petersen, Alan F. Wright, Steven G. Younkin, Kevin Morgan

**Affiliations:** 1 Department of Neuroscience, Mayo Clinic College of Medicine, Jacksonville, Florida, United States of America; 2 Medical Research Council (MRC) Human Genetics Unit, The Institute of Genetics and Molecular Medicine, Western General Hospital, Edinburgh, United Kingdom; 3 Department of Neurology, Mayo Clinic College of Medicine, Jacksonville, Florida, United States of America; 4 Department of Public Health Sciences, University of Edinburgh Medical School, Edinburgh, Scotland, United Kingdom; 5 Croatian Centre for Global Health, University of Split Medical School, Split, Croatia; 6 Centre for Clinical Medical Research, University Hospital “Sestre Milosrdnice”, Zagreb, Croatia; 7 School of Molecular Medical Sciences, Institute of Genetics, Queen's Medical Centre, University of Nottingham, Nottingham, United Kingdom; 8 Division of Psychiatry and Neuroscience, School of Medicine and Dentistry, Queen's University Belfast, Belfast, Northern Ireland, United Kingdom; 9 Department of Clinical Science at North Bristol, University of Bristol, Frenchay Hospital, Bristol, United Kingdom; 10 Greater Manchester Neurosciences Centre, University of Manchester, Manchester, United Kingdom; 11 Oxford Project to Investigate Memory and Ageing (OPTIMA), University Department of Physiology, Anatomy and Genetics, Oxford, United Kingdom; 12 Memory Assessment and Research Centre, University of Southampton, Southampton, United Kingdom; 13 Division of Neuroscience, University of Birmingham, Birmingham, United Kingdom; 14 Department of Psychiatry, University of Bonn, Bonn, Germany; 15 Department of Biostatistics, Mayo Clinic and Mayo Foundation, Rochester, Minnesota, United States of America; 16 Department of Neurology and the Mayo Alzheimer Disease Research Center, Mayo Clinic College of Medicine, Rochester, Minnesota, United States of America; National Institutes of Health, United States of America

## Abstract

**Background:**

The insulin-degrading enzyme gene (*IDE*) is a strong functional and positional candidate for late onset Alzheimer's disease (LOAD).

**Methodology/Principal Findings:**

We examined conserved regions of *IDE* and its 10 kb flanks in 269 AD cases and 252 controls thereby identifying 17 putative functional polymorphisms. These variants formed eleven haplotypes that were tagged with ten variants. Four of these showed significant association with *IDE* transcript levels in samples from 194 LOAD cerebella. The strongest, rs6583817, which has not previously been reported, showed unequivocal association (p = 1.5×10^−8^, fold-increase = 2.12,); the eleven haplotypes were also significantly associated with transcript levels (global p = 0.003). Using an *in vitro* dual luciferase reporter assay, we found that rs6583817 increases reporter gene expression in Be(2)-C (p = 0.006) and HepG2 (p = 0.02) cell lines. Furthermore, using data from a recent genome-wide association study of two Croatian isolated populations (n = 1,879), we identified a proxy for rs6583817 that associated significantly with decreased plasma Aβ40 levels (ß = −0.124, p = 0.011) and total measured plasma Aβ levels (b = −0.130, p = 0.009). Finally, rs6583817 was associated with decreased risk of LOAD in 3,891 AD cases and 3,605 controls. (OR = 0.87, p = 0.03), and the eleven *IDE* haplotypes (global p = 0.02) also showed significant association.

**Conclusions:**

Thus, a previously unreported variant unequivocally associated with increased *IDE* expression was also associated with reduced plasma Aß40 and decreased LOAD susceptibility. Genetic association between LOAD and *IDE* has been difficult to replicate. Our findings suggest that targeted testing of expression SNPs (eSNPs) strongly associated with altered transcript levels in autopsy brain samples may be a powerful way to identify genetic associations with LOAD that would otherwise be difficult to detect.

## Introduction

Late onset Alzheimer's disease (LOAD) is the most common cause of dementia in the elderly affecting approximately 10% of those over the age of 65 years. LOAD is a neurodegenerative condition in which the brain develops large numbers of extracellular senile plaques composed primarily of aggregated amyloid-ß proteins (Aß) as well as intraneuronal neurofibrillary tangles composed mainly of aggregated tau protein [1]. Mutations in the *APP*, *PSEN1*, and *PSEN2* genes that increase production of Aß are known causes of the rare, early onset, familial form of AD (for review see ref. [2]). Twin studies indicate that genetic factors are also important in late onset AD and account for at least 60% of its phenotypic variance [3], [4]. The *APOE* ε4 allele, which is present in ∼50% of AD cases, is associated with a substantial increase in the risk of LOAD, but it is neither necessary nor sufficient for the development of AD [5]–[7]. Despite an intense search in the past decade, identification of the additional genes that contribute to phenotypic variance in LOAD has proven difficult. Promising new LOAD genes such as *SORL1* [8], *GAB2* [9], *PCDH11X* [10], *PICALM* [11], *CLU* [11], [12] and *CR1* [11], [12] are, however, now being identified through genome-wide association studies and follow-up analyses in large case-control series. Here, we evaluate variants in *IDE* for association with LOAD and with two additional endophenotypes that have important functional implications.

*IDE* is a strong functional LOAD candidate gene because it encodes the insulin degrading enzyme, which has been shown to degrade Aß [13]–[17] and to influence brain Aß levels *in vivo* [18]–[20]. *IDE* has also obtained much attention as an LOAD candidate gene because of its location near LOAD linkage peaks. The *IDE* gene is located on chromosome 10q23.33, very near a ‘suggestive’ linkage peak (chromosome 10q24) found in two genome-wide linkage studies of LOAD families [21], [22] and a ‘significant’ linkage peak (10q) in a third [23]. The latter study showed allele-specific association of a variant (D10S583) within 195 kb of *IDE* with LOAD, an association that was confirmed by independent follow-up analysis in an LOAD case-control series [24]. Independent haplotype analysis by Prince et al. of a 276-kb block encompassing *IDE* showed highly significant association with LOAD and with several intermediate LOAD phenotypes in multiple case-control series [25]. When we performed follow-up analysis on these haplotypes [26], we found highly significant association with LOAD in one case-control series and significant association with plasma Aβ42 levels in extended LOAD families. These findings were supported by an independent study that associated *IDE* variants with Aβ plaque density and cognitive function in three independent Swedish LOAD series and in a follow-up English series [27], although the variants were not directly associated with disease risk.

Many *IDE* variants, including those studied by Prince et al. and by our group, have been analyzed in additional follow-up studies of Caucasian [27]–[38], Japanese [39], and Han Chinese [40] case-control series. Some of these studies showed significant evidence for association [29], [30], [33], [34], [36], [40] whereas others did not [27], [28], [31], [32], [35], [37]–[39]. AlzGene (www.alzforum/alzgene) meta-analysis of 12 variants from 21 publications investigating *IDE* to date shows no significant association.

Overall, studies of *IDE* variants provide evidence for association with LOAD that is difficult to dismiss as a set of false positives, but the association is not compelling because replication is inconsistent and the results of meta-analyses do not achieve significance [41].

## Results

Using dHPLC followed by sequencing, we screened conserved regions (≥70% identity identity in human vs rodent sequence) in the *IDE* gene and in 10 kb of 5′ and 3′ flanking sequence for variants likely to be functional. In each of the 521 subjects screened (269 cases and 252 controls), we evaluated 40 amplicons that contained a total of 9,501 bp of conserved DNA. This DNA included all exons (5,954 bp), conserved regions in the 5′ (268 bp) and 3′ (1,465 bp) flanking regions, and 1,814 bp of highly conserved intronic sequence. This search (**Table S1** online) yielded 17 common polymorphisms (minor allele frequencies >0.01) likely to have functional effects (**Table S2** and **Figure S1** online). These variants and the haplotypes they form were tested for association with *IDE* mRNA expression using transcript levels measured in 194 LOAD cerebella sampled at autopsy from the brain bank at Mayo Clinic, Jacksonville. They were also analyzed for genetic association with LOAD in one of the largest LOAD case-control series studied to date, which consisted of 3,529 AD cases and 3,441 controls drawn from four American and five North European series.

**Figure 1:**
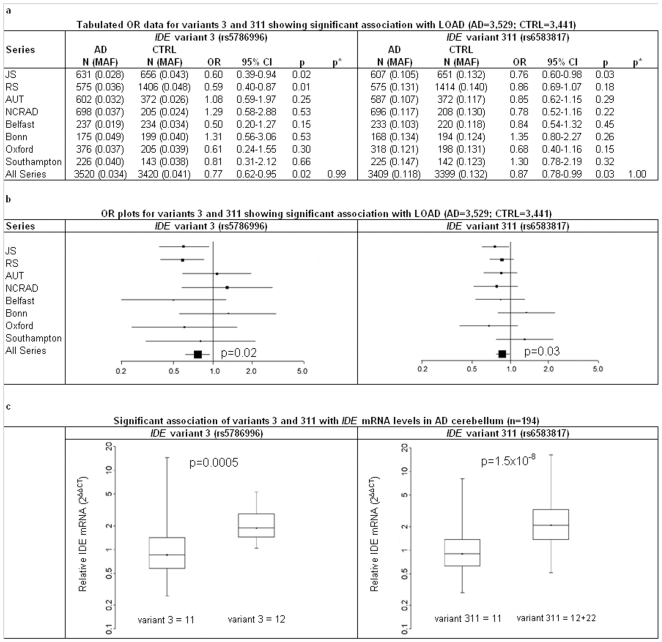
Association of variants 3 (rs5786996) and 311 (rs6583817) with LOAD and *IDE* mRNA levels. (**A**) Variants were analyzed for association with LOAD using an additive logistic regression model with age at diagnosis/entry, sex and *APOE* ε4 dosage as covariates. N; total genotype count, MAF; minor allele frequency. For logistic regression of the combined series, individual series were included as covariates. P values demonstrating that there were no series-related effects on association (p*) were obtained by comparing this model to one that adjusts for series x genotype interactions in addition to age at diagnosis/entry, sex, *APOE* ε4 dosage, and series effects. (**B**) OR and 95% CI forest plots were generated from the data tabulated in panel a. (**C**) Association of the two variants with *IDE* mRNA levels in cerebellum. The horizontal black line and dots represent median relative *IDE* mRNA level expressed as 2^−ΔΔCt^ for each variant. Boxes represent the 25th and 75th percentiles and whiskers represent the full data range.11 = homozygotes for the major allele, 12 = heterozygotes, 22 = minor allele homozygotes. Samples with 12 and 22 genotypes for rs6583817 were combined because only two samples were 22. Variants were analyzed for association with the level of *IDE* transcript (ΔCT) using an additive linear regression model with age at diagnosis/entry, sex and *APOE* ε4 dosage as covariates.

The 17 *IDE* variants formed eleven haplotypes with frequencies >1% that could be tagged with ten variants (**Tables S3** and **S4** online). In the combined series, these haplotypes (**Table 1**) were significantly associated with *IDE* transcript levels (global p = 0.003) and with LOAD (global p = 0.02). Of the ten tagging variants, four showed significant association with the level of *IDE* transcript (**Table 2**). Association was most significant for v311 (rs6583817) and v3 (rs5786996) with p-values of 1.5×10^−8^ and 0.0005 respectively, and linear regression showed that for both of these variants the average level of *IDE* transcript was more than 2-fold higher for each minor allele carried. In the combined series, the minor alleles of v311 (p = 0.03) and v3 (p = 0.02) were also associated with reduced risk of LOAD. Notably, neither variant has previously been analyzed for association with LOAD, so they are not included in the AlzGene database. The association of these two variants with LOAD showed no series-related effects. Results for each of the eight series that were tested are shown in **Figure 1A and 1B**. **Figure 1C** shows box plots which compare the relative level of *IDE* transcript in carriers of these two variants as compared to non-carriers.

**Table 1:** Association of *IDE* common conserved haplotypes with cerebellar *IDE* mRNA and LOAD.

Htype	Freq	Variant ID										Association with *IDE* mRNA (n = 194 autopsy LOAD cerebellum; global p = 0.003)			Association with LOAD (LOAD = 3,529, CTRL = 3,441; global p = 0.02)	
		2	3	6	154	310	311	176	46	684	685	β	Fold Δ (95% CI)	p	OR (95% CI)	p
H1	0.24	0	0	0	0	0	0	0	0	0	0	0.08	0.94 (0.75–1.19)	0.62	0.95 (0.86–1.05)	0.32
H2	0.19	0	0	0	0	0	0	0	0	0	1	0.34	0.79 (0.64–0.98)	**0.03**	1.09 (0.98–1.21)	0.13
H3	0.1	0	0	0	1	0	0	1	1	0	0	0	1.00 (1.00–1.00)	0.85	0.94 (0.82–1.08)	0.40
H4	0.11	0	0	1	0	0	0	0	0	0	0	−0.16	1.11 (0.77–1.61)	0.56	0.97 (0.85–1.11)	0.64
H5	0.1	0	0	0	1	0	0	0	1	0	0	0	1.00 (1.00–1.00)	0.49	1.23 (1.06–1.41)	**0.005**
H6	0.09	0	0	0	1	0	1	0	0	0	0	−1.16	2.24 (1.56–3.21)	**1.0×10^−5^**	0.89 (0.77–1.03)	0.11
H7	0.05	0	0	0	0	0	0	0	1	0	0	−0.17	1.12 (0.75–1.67)	0.57	1.11 (0.91–1.35)	0.32
H8	0.05	0	0	0	0	0	0	0	0	1	0	0.12	0.92 (0.60–1.41)	0.71	0.89 (0.73–1.08)	0.25
H9	0.04	0	1	0	1	0	1	0	0	0	0	−1.22	2.34 (1.28–4.27)	**0.006**	0.79 (0.63–0.99)	**0.04**
H10	0.02	1	0	0	1	0	0	0	0	0	0	0.19	0.88 (0.47–1.63)	0.68	1.26 (0.95–1.68)	0.11
H11	0.02	0	0	0	0	1	0	0	0	0	0	0.25	0.84 (0.45–1.56)	0.58	1.14 (0.82–1.58)	0.44

Haplotypes are numbered according to their frequency. Only haplotypes with frequency >0.01 are shown. The “Haplotype” and “Variant ID” columns show the allelic composition of each haplotype in the 5′ to 3′ orientation from the p to the q telomere of chromosome 10. The allelic composition of each haplotype is depicted as “0” for no minor allele and “1” for minor allele. Freq = haplotype frequency in the combined series. Haplotype linear regression analyses vs. *IDE* mRNA levels were performed using an additive model adjusting for *APOE* ε4 dosage, age at diagnosis/entry and sex. β = regression coefficient. Fold difference in expression is derived from the term (2^(−β)^). 95% confidence intervals (CI) were derived from the term 2^[−(β±SEM)]^; p = p value. Haplotype logistic regression analyses vs. LOAD were carried out using an additive model adjusting for *APOE* ε4 dosage, age at diagnosis/entry, sex and series. OR = odds ratio.

**Table 2:** Association of *IDE* common conserved variants with cerebellar *IDE* mRNA and LOAD.

Variant details									Association with IDE mRNA (n = 194 LOAD autopsy cerebellum)			Association with LOAD (LOAD = 3,529; CTRL = 3,441)			
ID	rs number	Position	Location	Cons.	MAF	HWE-p	1	2	β	Fold Δ (95% CI)	p	AD	CTRL	OR (95% CI)	p
2	N/A	94,202,383	3′ flank	81%	0.02	0.15	AA	–	−0.03	1.02 (0.60–1.74)	0.94	0.02	0.02	1.15 (0.88–1.50)	0.30
3	rs5786996	94,202,516	3′ flank	74%	0.04	0.04	-	C	−1.08	2.12 (1.40–3.21)	**0.0005**	0.03	0.04	0.77 (0.62–0.95)	**0.02**
6	rs5786997	94,203,071	3′ flank	88%	0.11	0.93	–	AT	−0.09	1.07 (0.78–1.45)	0.69	0.1	0.11	0.96 (0.84–1.09)	0.52
154	rs4646957	94,219,892	Intron18	71%	0.36	0.88	G	A	−0.28	1.22 (1.03–1.43)	**0.02**	0.35	0.36	0.98 (0.90–1.07)	0.67
310	N/A	94,236,972	Exon13	92%	0.08	1	G	T	−0.22	1.16 (0.68–1.98)	0.58	0.02	0.02	1.09 (0.80–1.48)	0.60
311	rs6583817	94,237,227	Intron12	74%	0.13	0.6	G	A	−1.08	2.12 (1.65–2.71)	**1.5×10^−8^**	0.12	0.13	0.87 (0.78–0.99)	**0.03**
176	rs17875327	94,264,789	Intron4	85%	0.1	0.01	T	C	0.18	0.88 (0.67–1.15)	0.35	0.1	0.10	0.97 (0.84–1.10)	0.61
46	rs4646955	94,284,271	Intron3	76%	0.25	0.14	T	C	0.10	0.93 (0.78–1.12)	0.46	0.26	0.25	1.07 (0.98–1.17)	0.14
684	rs17107721	94,288,480	Intron1	71%	0.05	0.72	G	A	−0.07	1.05 (0.73–1.50)	0.8	0.05	0.05	0.88 (0.73–1.06)	0.18
685	rs11187061	94,295,389	Intron1	70%	0.18	0.57	C	T	0.35	0.78 (0.65–0.94)	**0.009**	0.19	0.18	1.07 (0.96–1.18)	0.20

Variant IDs and rs numbers (dbSNP variant identifier) are given for the 10 tagging variants; Chr position indicates relative position to the Human Genome build 36.1. Cons = conservation (≥70% identity between the human and mouse sequence); MAF = minor allele frequency in controls; HWE-p = Hardy-Weinberg Equilibrium p value in controls; columns labeled as 1 and 2 indicate the major and minor allele, respectively. Single variant linear regression analyses vs. *IDE* mRNA levels were performed using an additive model adjusting for *APOE* ε4 dosage, age at diagnosis/entry and sex. β = regression coefficient. Fold difference in expression due to a single copy of the minor allele is derived from the term (2^(−β)^). 95% confidence intervals (CI) were derived from the term 2^[−(β±SEM)]^; p = p value. Single variant logistic regression analyses vs. LOAD were carried out using an additive model adjusting for *APOE* ε4 dosage, age at diagnosis/entry, sex and series. OR = odds ratio.

Additional discussion of the complex single variant and haplotype associations shown in **Table 1** and **Figure 1** is provided in the **Text S1** online. There, we consider two contrasting models; a simple model where v311 is postulated to be the only variant that influences *IDE* function and a more complex model where multiple haplotypes would have independent effects. We emphasize that the current study has inadequate power to distinguish between these models.

We further explored the involvement of *IDE* in LOAD pathogenesis by evaluating the effect of *IDE* on Aß levels. To this end, we analyzed plasma Aß40, Aß42 and total Aß (Aß40 +Aß42) in 949 individuals from Vis and 930 individuals from Korcula (total = 1,879), which are two isolated populations from the Dalmatian islands recently analyzed for multiple disease related traits using genome-wide scans [42]–[44].

Our analysis took advantage of the strong linkage disequilibrium between v311 and rs7910977 (r2 = 0.996 and D′ = 1), since rs7910977, but not v311, was included in the genome-wide scan. This analysis (**Table S5** online) showed that rs7910977 was significantly associated with reduced levels of plasma Aß40 in the Vis population (ß ± SE = −0.147±0.06 p = 0.018) and suggestively associated with reduced Aβ40 in the Korcula population (ß ± SE = −0.086±0.08, p = 0.27). Meta-analysis of the two populations showed overall significant association with reduced Aß40 (ß ± SE = −0.124±0.05, p = 0.011). Analysis of the two populations (**Table S5** online) also showed a suggestive association between rs7910977 and reduced plasma Aß42 levels (meta-analysis ß ± SE = −0.07±0.05, p = 0.18). Similarly, analysis of total Aß (Aß40 +Aß42) revealed significant association with reduced levels in the Vis population (ß ± SE = −0.165±0.06, p = 0.011) and meta-analysis of the two populations showed overall significant association with reduced total Aß (ß ± SE = −0.130±0.05, p = 0.009) Thus the variant that showed strongest association with elevated *IDE* expression also showed significant association with reduced levels of Aß, a substrate of the insulin degrading enzyme that is implicated in LOAD pathogenesis.

**Figure 2:**
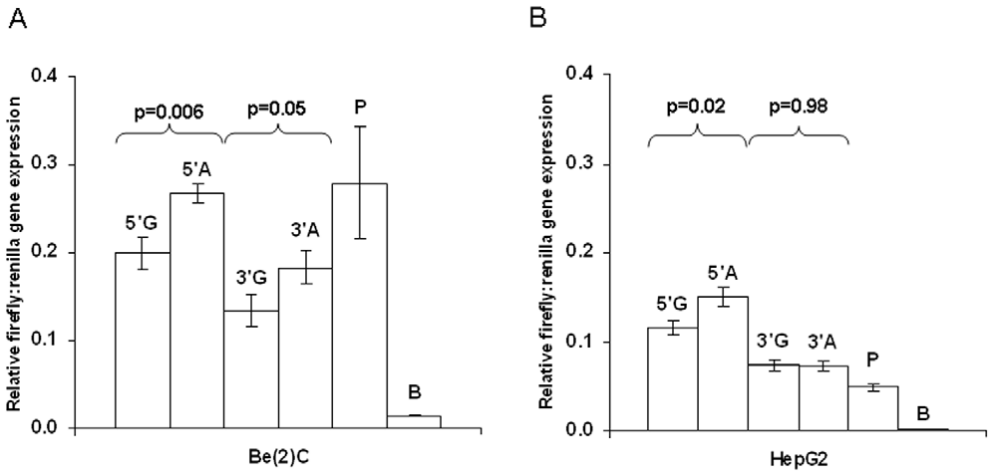
*In vitro* functional effects of variant 311 (rs6583817) on reporter gene expression. pGL3P constructs containing v311 sequence were transfected into (**A**) Be(2)-C and (**B**) HepG2 cell lines. 5′ and 3′ refer to the position of the cloned sequences relative to the luciferase gene and promoter in the pGL3P vector. G refers to vectors containing v311 major allele sequence and A refers to vectors containing v311 minor allele sequence. P refers to the pGL3P positive control vector (containing SV40 promoter) and B refers to the pGL3B negative control vector (containing no promoter). Error bars represent SEM (standard error of the mean). Note that the major allele sequence encompassing v311 had a repressor effect in Be(2)-C cells that achieved significance when the 3′G construct was compared to the pGL3P positive control vector (P) alone (fold-change = 0.48, p = 0.04). In contrast, the wild type sequence had a significant enhancer effect when the 3′G construct was compared to pGL3P in HepG2 cells (5′G fold-change = 2.4, p = 0.02, 3′G fold-change = 1.5). This difference probably reflects differential regulation of *IDE* expression by the transcription factors expressed in each cell line.

The analysis described in our **Text S1** online indicates that our results could all be accounted for by a simple model where v311 is the only variant that influences *IDE* function. For this reason and because v311 showed the strongest association with increased level of *IDE* transcript, we evaluated its functional effect on reporter gene expression *in vitro* (unlike rs7910977, v311 is located in a conserved region of *IDE* and is therefore more likely to be a functional variant). Two immortalized human cell lines (Be(2)-C neuroblastoma and HepG2 hepatocytoma) were transfected with expression constructs containing *IDE* sequence surrounding v311. Major (G) and minor (A) allele sequences were cloned either 5′ to the promoter or 3′ to the gene. In neuroblastoma (Be(2)-C) cells (**Figure 2A**), transfection of the 5′A (minor allele) construct significantly increased reporter gene expression compared to the 5′G (major allele) construct (5′A vs 5′G = 1.35-fold increase in reporter expression, p = 0.006, n = 5). This result replicated well in HepG2 hepatocellular carcinoma cells (**Figure 2B**: 5′A vs 5′G = 1.31-fold increase in reporter expression, p = 0.02, n = 3). In Be(2)-C cells the same allele-specific reporter expression was observed for the 3′ constructs (3′A vs 3′G fold-increase = 1.35, p = 0.05), but this was not apparent in HepG2 cells (3′A vs 3′G fold-change = 0.98, p = 0.44). This analysis of reporter gene expression focuses on the effect of the v311 locus in two cell lines where regulation of *IDE* expression is likely to be different than in human cerebellum. The results of this analysis do, nonetheless, provide useful supporting evidence that v311 is a functional variant that acts to increase *IDE* gene expression thereby decreasing Aß and decreasing risk for LOAD.

Variant 3 (v3) is in near perfect linkage disequilibrium with v311 but it occurs less frequently that v311. Thus v3 tags only one of the two haplotypes that include v311 (H9 but not H6), and both haplotypes show essentially identical association with the level of *IDE* transcript in brain (**Table 1**). Our point estimates of ORs for the association of v3 and v311 with LOAD (**Table 2**) suggest that v3 may have a stronger protective effect than v311, but the 95% CIs for the ORs of these two variants are completely overlapping. Thus we have insufficient power to determine whether the protective effects of the two variants are truly different, and the association of v3 with increased *IDE* transcript and reduced risk of LOAD could be due entirely to a functional effect of v311.

**Figure 3:**
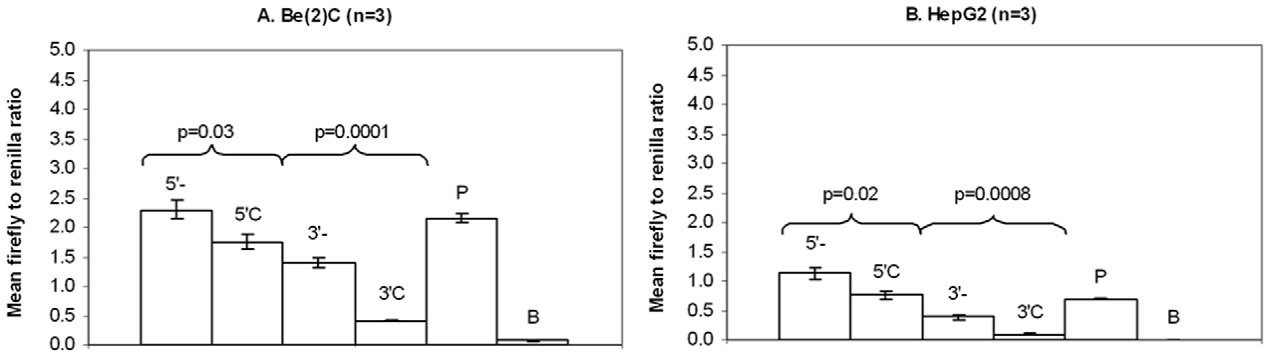
*In vitro* functional effects of variant 3 (rs5786996) on reporter gene expression. Note that variant 3 (-/C) has a single C insertion. Thus the major allele is indicated by a – and the minor allele by a C. pGL3P constructs containing v3 sequence were transfected into (**A**) Be(2)-C and (**B**) HepG2 cell lines. 5′ and 3′ refer to the position of the cloned sequences relative to the luciferase gene and promoter in the pGL3P vector. “-” = vector containing v3 major allele sequence, “C” = vector containing v3 minor allele insertion sequence, “P” = pGL3P positive control vector (containing SV40 promoter), “B” = pGL3B negative control vector (containing no promoter). Error bars represent standard error of the mean. Note that in Be(2)-C cells, the v3 sequence had a repressor effect when compared to the pGL3P positive control vector (P) alone that achieved significance (5′C; fold-change = 0.82, p = 0.03, 3′-; fold-change = 0.65, p = 0.001; 3′C; fold-change = 0.19, p<0.0001) for all except the 5′- major allele sequence that showed no significant change in expression. In HepG2 cells, the 5′major allele sequence had an enhancer effect (5′-; fold-change = 1.62, p = 0.008) while the 5′C vector showed no significant change in expression compared to P. Both 3′ constructs had repressor activity (3′- fold-change = 0.56, p = 0.0008, 3′C fold-change = 0.14, p<0.0001).

To determine if v3 might also contribute functionally to increased *IDE* expression, we evaluated its functional effect on reporter gene expression in vitro. Two immortalized human cell lines (Be(2)-C neuroblastoma and HepG2 hepatocytoma) were transfected with expression constructs containing 248bp of *IDE* sequence surrounding v3. In Be(2)-C cells (**Figure 3A**), transfection of the minor allele (C) construct significantly decreased reporter gene expression compared to the major allele (-) construct in both 5′ and 3′ locations (5′C vs 5′-; fold-change = 0.77, p = 0.03, 3′C vs 3′-; fold-change = 0.29, p = 0.0001). This result was replicated in HepG2 cells (**Figure 3B**: 5′C vs 5′-; fold-change = 0.67, p = 0.02, 3′C vs 3′-; fold-change = 0.26, p = 0.0008).

These results showing that v3 is associated with decreased expression in vitro make it unlikely that v3 functions to increase *IDE* expression in brain and suggest that the association of v3 with increased transcript in brain and reduced risk of LOAD may be due to its strong linkage disequilibrium with v311. These results underscore the limitation of pursuing SNP/transcript associations observed in human brain by analyzing reporter gene expression in cell lines where regulation may differ considerably from that in brain. The mechanism of the increased expression we observed in vitro and the implications, if any, for mechanisms operative in brain are uncertain.

## Discussion

We incorporated several elements into the design of this study that were intended to improve our ability to determine whether or not *IDE* has LOAD susceptibility alleles. First, we focused on variants in conserved regions. Like other markers, these variants can detect un-typed functional variants indirectly through linkage disequilibrium. They are advantageous because they are more likely to play a functional role in disease development than anonymous polymorphisms and are therefore expected to have higher power to detect genetic associations. Second, in an effort to detect the net effect of associations from multiple variants, we tested for global haplotypic association. Third, we tested these *IDE* haplotypes and variants for association with *IDE* gene expression levels. Fourth, we evaluated a large series of 3,529 cases and 3,441 controls to increase our power to detect weak associations with LOAD. Finally, we used results from a separate genome wide scan to test for association with the level of Aß40, Aß42 and the sum of the two, in plasma.

Because it is a single test, it is reasonable to minimize multiple testing of the 17 variants identified in conserved regions by beginning with global tests of haplotypic association. This approach was effective. Global haplotypic association with the level of *IDE* transcript was highly significant (p = 0.003), and global haplotypic association with LOAD was also significant (p = 0.02).

Testing for association with the level of *IDE* transcript was also useful at the single variant level. The variants showing strongest association were v311 (p = 1.5×10^−8^) and v3 (p = 0.0005). These significant, functionally meaningful associations provide a powerful rationale for prioritizing the testing of the 17 *IDE* variants for association with LOAD beginning with these two expression SNPs (eSNPs). When assessed in the LOAD case-control series, these variants did in fact show significant (p<0.03) association with reduced risk of LOAD.

It is likely that some LOAD susceptibility alleles alter gene function similarly in affected and unaffected brain regions and even in peripheral tissues and lymphoblastoid cell lines, whereas others alter gene function selectively in affected brain regions. In this study, we analyzed *IDE* mRNA in LOAD brain tissue obtained at autopsy to improve detection of LOAD-related *IDE* variant/transcript associations that might go undetected in peripheral tissues or lymphoblastoid cell lines. Because LOAD causes profound neuronal cell loss and astrogliosis that will alter mRNA levels in affected regions, mRNAs were measured in the cerebellum which is largely unaffected by LOAD pathology. This approach presupposes that at least some *IDE* variant/transcript associations relevant to LOAD can be detected not only in affected brain regions but also in cerebellum. mRNAs are degraded in the postmortem interval, and mRNA levels are likely to be influenced by the agonal state prior to death. These factors will alter *IDE* transcript level, but they can have a similar effect in carriers and non-carriers of functionally important variants that does not alter the difference in transcript level between carriers and non-carriers. This probably explains why we were able to detect unequivocal, functionally meaningful *IDE* variant/transcript associations using samples obtained at autopsy from only 194 subjects.

Collectively our findings provide the best evidence to date that *IDE* has susceptibility alleles for LOAD. More generally, they suggest that targeted testing of expression SNPs (eSNPs) strongly associated with altered transcript levels in brain samples frozen at autopsy may be a powerful way to identify genetic associations with CNS diseases that would otherwise be difficult to detect.

## Materials and Methods

### Ethics Statement

Approval was obtained from the ethics committee or institutional review board of each institution responsible for the ascertainment and collection of samples (Mayo Clinic College of Medicine, Jacksonville, FL and Mayo Clinic College of Medicine, Rochester, MN, USA; MRC Human Genetics Unit, Western General Hospital and the University of Edinburgh Medical School, Edinburgh, UK; University of Split Medical School and University Hospital “Sestre Milosrdnice”, Croatia; Queen's Medical Centre, Nottingham, UK; Queen's University Belfast, Northern Ireland, UK; University of Bristol, Frenchay Hospital, UK; University of Manchester, UK; University of Oxford, UK; and University of Bonn, Germany. Written informed consent was obtained for all individuals that participated in this study.

### Case-Control Subjects

The age-at-diagnosis/entry, diagnosis method, and percent female in the LOAD cases and controls for each USA and ART series are shown in **Table S1**.

### USA Case-Control Subjects

The USA case-control series consisted of 5,173 Caucasian subjects from the United States (2,513 AD, 2,660 control) ascertained at the Mayo Clinic (1,811 AD, 2,451 controls) or through the National Cell Repository for Alzheimer's Disease (NCRAD: 702 AD, 209 control). All subjects ascertained at the Mayo Clinic in Jacksonville, Florida (JS: 632 AD, 660 control) and at the Mayo Clinic in Rochester, Minnesota, (RS: 576 AD, 1,417 control) were diagnosed by a Mayo Clinic neurologist. The neurologist confirmed a Clinical Dementia Rating score of 0 for all JS and RS subjects enrolled as controls; cases had diagnoses of possible or probable AD made according to NINCDS-ADRDA criteria [45]. In the autopsy-confirmed series (AUT: 603 AD, 374 control), all brains were evaluated by Dr. Dennis Dickson and came from the brain bank maintained at the Mayo Clinic in Jacksonville, FL. In the AUT series the diagnosis of definite AD was also made according to NINCDS-ADRDA criteria. All AD brains analyzed in the study had a Braak score of 4.0 or greater. Brains employed as controls had a Braak score of 2.5 or lower but often had brain pathology unrelated to AD and pathological diagnoses that included vascular dementia, frontotemporal dementia, dementia with Lewy bodies, multi-system atrophy, amyotrophic lateral sclerosis, and progressive supranuclear palsy. One AD case from each of the 702 late-onset NCRAD families was analyzed. NCRAD AD cases were selected based on strength of diagnosis (autopsy-confirmed: 32%>probable: 45%>possible: 8%>family report: 15%); the case with the earliest age at diagnosis was taken when several cases had equally strong diagnoses. The 209 NCRAD controls that we employed were unrelated Caucasian subjects from the United States with a Clinical Dementia Rating of 0, specifically collected for inclusion in case-control series.

### Alzheimer's Research Trust (ART) Case-Control Subjects

Samples from a total of 1,797 subjects were obtained from five Alzheimer's Research Trust, UK (ART) network centers, as shown in **Table S1**. All samples were from subjects who were diagnosed clinically using NINCDS-ADRDA criteria [45]. All patients with evidence of an autosomal dominant AD trait, or where a first degree relative had been diagnosed with familial AD, were excluded. Since there were no controls available for the Manchester series, the Manchester AD samples were combined with those in the Oxford series when the individual series were analyzed.

### DNA Isolation

For the JS and RS samples, DNA was isolated from whole blood using an AutoGen instrument (AutoGen, Inc, Holliston, MA). The DNA from AUT samples was extracted from cerebellum using Wizard® Genomic DNA Purification Kits (Promega Corp., Madison, WI). DNA from the RS and AUT series was scarce, so samples from these two series were subjected to whole genome amplification using the Illustra GenomiPhi V2 DNA Amplification Kit (GE Healthcare Bio-Sciences Corp., Piscataway, NJ).

For the ART samples, genomic DNA was extracted from whole blood samples or brain tissue using the QIAamp DNA blood mini kit (Qiagen, Crawley, West Sussex, UK).

### Variant Discovery

Conserved segments in the region encompassing all 25 exons of *IDE* and up to 10 kb from the 5′ and 3′ ends of the gene, were screened for DNA variation likely to be functional in a subset of the JS series (269 cases and 252 controls). This sample size gave us enough power to detect all polymorphisms that occur at a frequency >1% in this population. The criteria used for conservation was a cut-off of ≥70% identity over 100 bp windows between the human and mouse sequence or between the human and rat sequence, as determined by the pre-computed alignments in the VISTA Genome Browser (http://pipeline.lbl.gov/cgi-bin/gateway2). The human genome position screened was chr10:94,335,255-94,475,049 in the Human April 2003 genome build. The latter build was used as the base genome, and was compared to the Mouse Feb. 2003 and Rat Jan. 2003 builds.

In each of the 521 subjects screened, we evaluated 40 amplicons that contained a total of 9,501 bp of conserved DNA. This DNA included all exons (5,954 bp) as well as conserved regions located up to 10kb from the gene's 5′ end (268 bp), up to 10kb from the gene's 3′ end (1,465 bp), and 1,814 bp of highly conserved intronic sequence. Forty PCR primer pairs were designed to screen the targeted conserved segments via denaturing high performance liquid chromatography (dHPLC). PCR amplicons were generated with 20ng of DNA in a 50ul PCR containing 0.2µM forward primer, 0.2µM reverse primer, 200µM dNTPs, 5ul of 10× reaction buffer with 25mM MgSO_4_ (Transgenomic, Inc.), and 1 Unit of Optimase® Polymerase (Transgenomic, Inc.), using one of the following three conditions in a Hybaid thermocycler: 60–50 Touchdown, 62–57 Touchdown, or 55–45 Touchdown. Each PCR product was denatured at 95°C for 10 min and cooled slowly to 25°C at a rate of 0.03°C/sec to encourage heteroduplex formation. 5µl of each sample was injected into a DNASep® HT Cartridge 6.5mm×37mm (Transgenomic, Inc.) and analyzed in a WAVE DHPLC instrument (Transgenomic, Inc.) to identify heterozygotes. The optimal oven temperature and WAVE Optimized® buffer gradient for DHPLC analysis of each amplicon was selected using the Navigator™ software (Transgenomic, Inc.). Samples were categorized as either heteroduplexes or homoduplexes, based on the resulting elution profiles as recommended by Transgenomic, Inc. Representative homoduplexes and heteroduplexes from each amplicon were sequenced in order to determine the nature of the DNA variation underlying each heteroduplex profile. 20µl of remaining PCR product from the selected samples were purified for the sequencing reaction using the MultiScreen® PCR96 Filter Plates (Millipore). Sequencing in the forward and reverse orientation was performed at the Molecular Biology Core Facility at the Mayo Clinic, Rochester, MN as described on their website (http://mayoweb.mayo.edu/mbcf/mbcf/dna_seq.html). This variant discovery effort yielded 53 variants (42 novel, as shown in **Table S2**) of which 14 had minor allele frequencies (MAFs) >1%. Twelve of these were genotyped, 2 could not be converted into TaqMan assays (deep intronic, 1 base insertions within simple repeats).

To complement our *de novo* search for conserved variants, we developed a web based application called SNPMiner to mine known variants in conserved regions. As input the application takes a standard NCBI query string and/or a set of chromosomal coordinates. In real time, the application collects information from dbSNP (http://www.ncbi.nlm.nih.gov/projects/SNP/) on all variants that satisfy the input. Using VISTA's pre-computed human vs. mouse sequence alignments, which have been downloaded into our local server, SNPMiner scans all 100 bp regions encompassing each variant retrieved from dbSNP and identifies the 100 bp region with maximum identity between the mouse and human sequence. In this fashion, we identified all known variants in the *IDE* gene or its 10 kb flanks that were in conserved regions with human vs. mouse sequence identity >70%. The variants in conserved regions that had MAF information in dbSNP were genotyped, as described below in the section entitled “**Genotyping of** ***IDE*** **variants**”, in at least the Mayo JS series to determine if they met the MAF cut-off of ≥0.01. In the case of variants that did not have MAF information in dbSNP, dHPLC was used as described above to estimate their frequency. In the JS series, HaploView [46] was employed to determine the degree of linkage disequilibrium between the variants identified through our de novo search that could be genotyped with TaqMan assays.

Our review of all known *IDE* variants identified 15 additional variants, each located in a 100 bp region that had sequence identity of ≥70% between the mouse and human sequence. Of these, 8 had MAFs≥1%. Three of these were in perfect LD with variants from the de novo search, thus were eliminated (Variant IDs 925, 931 and 934, **Table S2**) leaving 17 common *IDE* variants in conserved regions to be genotyped. The location of the 68 *IDE* variants in conserved regions is shown relative to gene structure in **Figure S1a**. The locations of the 17 common variants that were genotyped are shown in **Figure S1b**. All 68 conserved variants are fully described in **Table S2**, where the 17 variants that were genotyped are shown in boldface type.

### Genotyping of *IDE* Variants

Variants found in conserved regions via dHPLC and those found in dbSNP having known MAFs were selected for genotyping. Conserved variants in dbSNP without known MAF were genotyped only if they were observed at least once via dHPLC analysis of 182 subjects. All genotyping of USA series samples was performed at the Mayo Clinic in Jacksonville using TaqMan® SNP Genotyping Assays in an ABI PRISM® 7900HT Sequence Detection System with 384-Well Block Module from Applied Biosystems, California, USA. The genotype data was analyzed using the SDS software version 2.2.2 (Applied Biosystems, California, USA).

The ART data, was also generated with TaqMan assays performed at Geneservice (Cambridge, UK). Fifteen percent of the samples assayed were of known genotype, determined by sequencing, which were unknown to Geneservice, but known at source and 10% were genotyped in duplicate as a quality assurance measure. The data were only accepted when there was 100% concordance between duplicate samples.

Details of the variants investigated in this study, including location, MAF, Hardy-Weinberg equilibrium p values and genotype counts can be found in **Table S3**.

### *IDE* Haplotypes

Analysis by HaploView [46] (solid spine of LD) showed that the 17 *IDE* variants were in strong linkage disequilibrium (**Figure S1c**). These variants formed 11 haplotypes (**Table S4**) with a frequency of 1.0% or more that could be identified with 10 tagging variants. Analysis by Haplo.Stats identified the same 11 haplotypes, which accounted for 96% of the *IDE* genetic variation captured by the 17 variants. Haplotype frequencies were estimated using the expectation-maximization approach implemented in the haplo.em function of haplo.stats v1.2.2 [47]. Haplo.stats was also used to create files showing the inferred haplotypes for each individual, which were used to perform logistic regression analyses.

### Measurement of *IDE* mRNA Expression

Total RNA was extracted from samples of cerebellum from 200 AD brains using an ABI PRISM 6100 Nucleic Acid PrepStation and the Total RNA Isolation Chemistry kit from Applied Biosystems. RNA was reverse transcribed to single-stranded cDNA using the High-Capacity cDNA Archive Kit from Applied Biosystems. Real-time quantitative PCR was performed in triplicate for each sample using ABI TaqMan Low Density expression Arrays (384-Well Micro Fluidic Cards) with a pre-validated TaqMan Gene Expression Assay. 18s ribosomal RNA (18s rRNA) was used as the endogenous control for the relative quantification of *IDE* mRNA. Real-time PCR cycle threshold (C_T_) raw data was collected and exported using the ABI PRISM® SDS software version 2.2.

The variable C_T_ within the raw data file indicates the PCR cycle number at which the amount of amplified gene target reaches a fixed threshold. The variable ΔC_T_ denotes the difference between the averaged C_T_ values for the *IDE* transcript and that for the reference 18S rRNA transcript.

The ΔC_T_ values calculated from each sample were used as quantitative phenotypes to determine associations between *IDE* variants and the level of *IDE* transcript. Some samples had one or more replicate measurements that failed to amplify and were obvious outliers, thus they were excluded from the analysis. All samples included for analysis had at least two replicates with a SEM ≤0.35.

### Association of *IDE* Haplotypes and Variants with the Level of *IDE* Transcript

The fold difference in the expression levels for the minor allele was estimated from the regression coefficient (ß) for the additive model that was fit to the ΔC_T_ data, which were expressed on the log_2_ scale, while also adjusting for *APOE* ε4 dosage, age at diagnosis/entry and sex. Therefore, we summarized the associations using 2^(−ß)^ to estimate the fold difference in expression due to a single copy of the minor allele, and 2^[−(ß±2SEM)]^ to estimate the corresponding 95% confidence interval (CI). We further summarized the expression differences graphically by preparing box plots showing the relative amount of *IDE* transcript in any sample in the series as compared to the mean expression level of all of the samples that were major homozygotes. This was done by computing the mean of all ΔC_T_ values for the samples in the major homozygote category and subtracting it from each of the ΔC_T_ values for all samples in all genotype categories (i.e. major homozygotes, heterozygotes and minor homozygotes) to obtain ΔΔC_T_ values. Given that these ΔΔC_T_ values are the log_2_ fold changes between two measurements, the relative expression levels between any sample and the mean of the major homozygotes is given by 2^−ΔΔCT^  =  2̂(−(ΔC_T_Any Sample_−mean(ΔC_T_Major Homozygotes_))). These 2^−ΔΔCT^ values are plotted in the box plots.

The overall association between estimated haplotypes (frequency >0.01 in combined dataset) and the level of *IDE* transcript (ΔC_T_) was determined by multivariable linear regression using a haplotypic dosage model with sex, age at diagnosis/entry, and *APOE* ε4 dosage (0, 1 or 2 copies of the ε4 alelle) as covariates. The association between individual haplotypes and *IDE* transcript (ΔC_T_) was determined by linear regression using an allelic dosage model with sex, age at diagnosis/entry, and *APOE* ε4 dosage as covariates. The haplo.stats package was used to create the files containing the inferred haplotypes for each individual that were used to perform these analyses.

*IDE* variants were analyzed for association with the level of *IDE* transcript (ΔC_T_) using an additive/allelic dosage (11 = 0, 12 = 1, 22 = 2) linear regression model that included sex, age at diagnosis/entry, *APOE* ε4 dosage and series as covariates.

### Power to Detect Association between *IDE* Variants and the Level of *IDE* Transcript

In our AUT series, we obtained *IDE* transcript levels from 194 LOAD brains. Using power formulae for an ordinal test of significance across the three possible genotypes using linear regression, we computed the detectable effect size while setting the power at 80% for a 0.05 level test for selected minor allele frequencies. Although the identified variants were in high LD, we re-computed these estimates after correcting for the 17 tests performed. With the sample size available to us, and with the minor allele frequencies of the *IDE* variants, we had 80% power to detect differences in ΔC_T_ values at least 1.11, 0.48 and 0.30 standard deviations for variants with MAFs of 1.7%, 10.3% and 35.5%, respectively. After applying Bonferroni corrections for 17 variants, these detectable effect sizes climbed to 1.52, 0.65 and 0.41. Given that the standard deviation of the ΔC_T_ values is 1.13, these detectable effect sizes correspond to fold-differences between carriers and non-carriers of 2^1.13×effect^. For the tests performed at the nominal level, for example, we had 80% power to detect a 2^1.13×1.11^ = 2.39-fold per-copy effect for the most rare variant and a 1.29-fold per-copy effect for the most common variant.

### Association of *IDE* Haplotypes and Variants with LOAD

The overall association between haplotypes (frequency >0.01 in combined dataset) and LOAD was determined using the global score statistic implemented in the haplo.score function incorporated in the haplo.stats package [47] while adjusting for sex, age at diagnosis/entry, *APOE* ε4 dosage, and series effects. The association between individual haplotypes and LOAD was determined by entering the estimated haplotypes for the cases and controls into univariate logistic regression using a haplotypic dosage model that adjusted for sex, age at diagnosis/entry, *APOE* e4 dosage, and series as covariates.

*IDE* variants were analyzed for association with LOAD by logistic regression using PLINK software [48] with sex, age at diagnosis/entry, and *APOE* ε4 dosage as covariates. For logistic regression using data from the combined series, we also included indicator variables for the individual series as covariates. All logistic regression analyses were performed using the additive/allelic dosage model as the primary test of significance for each variant. To test for evidence of any series-related effects on these associations, we compared this model to one that also incorporated series x genotype interactions in addition to all previously mentioned covariates. This analysis showed no evidence for series-related effects on any of the associations.

### Power to Detect Association between *IDE* Variants and LOAD

We have assembled the largest case-control series for LOAD genetic association studies available to date. In our combined series, we obtained samples from 3,529 cases and 3,441 controls. The minor allele frequencies (MAFs) of the 17 *IDE* variants identified in the conserved regions of the gene ranged from 1.7% to 35.5% with a median of 10.3%. Using power for the Armitage test for trend [49], we computed the minimum odds ratios (ORs) greater than one, and the maximum ORs less than one, that could be detected in a sample of this size while setting the power at 80% for a 0.05 level test. Although the identified variants were in high LD, we re-computed these estimates after a Bonferroni correction for the 17 tests performed. With the sample size available to us, and with the minor allele frequencies of the *IDE* variants, we had 80% power to detect ORs of at least 1.40, 1.17 and 1.11, or no more than 0.66, 0.85 and 0.90, for variants with MAFs of 1.7%, 10.3% and 35.5%, respectively. After applying Bonferroni corrections for 17 variants, the ORs greater than one climbed to 1.54, 1.22 and 1.14 and the ORs less than one dropped to 0.57, 0.80 and 0.87.

### Linkage Disequilibrium between rs6583817 (v311) and rs7910977

We previously conducted a LOAD genome wide association study (GWAS) with 313,504 SNPs in 844 cases and 1,255 controls that were also analyzed for association with the 17 *IDE* variants in conserved regions [10]. HaploView 3.1 was used to calculate the extent of LD between these 17 variants and the GWAS SNPs located in *IDE* or its 100 kb flanking regions. This analysis showed that GWAS SNP rs7910977 was in strong linkage disequilibrium with v311 (r^2^ = 0.996 and D′ = 1) identified in the current study. The measurements of *IDE* transcript levels described in the “Measurement of *IDE* mRNA expression” section above included 174 cerebellar samples obtained at autopsy from subjects that were part of our LOAD GWAS. Consistent with the results we report for v311, rs7910977 associated strongly with the level of *IDE* transcript in these 174 samples with a p value of 2.7×10^−8^ that was significant at the genome-wide level. rs7910977 also showed significant association with LOAD in the 2,099 subjects analyzed in the GWAS (p = 0.005).

### Association of rs7910977 with Plasma Aβ40 and Aβ42

rs7910977 was genotyped as part of two genome-wide association studies of the Dalmatian island populates of Vis and Korcula, using Illumina genotyping platforms [42], [44]. Genotypes for this SNP were present (following quality control) for 883 of the 949 subjects with plasma Aβ measurements in the Vis study and 876 of the 930 subjects with plasma Aβ measurements in the Korcula study. Study samples from Vis and Korcula were collected as described previously [42], [44] and plasma levels of the Aß40 and Aß42 peptides were measured, in duplicate, using a well established sandwich ELISA system, also described elsewhere [50], [51]. Antibody pairs BNT77/BC05 were used to measure plasma Aβ42 in both populations, the BAN50/BA27 pair was used to measure Aβ40 in the Vis population and the BNT77/BA27 pair was used to measure Aβ40 in the Korcula population. The ELISA assays were repeated for individuals that had a coefficient of variation (CV) of greater than 0.2 for the duplicate measurements; following repetition of these measurements the median value was used as the estimate of the plasma Aβ value. A third trait was created termed “total Aß” by summing these raw values for Aß40 and Aß42 for each person. This trait was then analysed in the same manner as the Aß40 and Aß42 traits individually.

The R package GenABEL [52] was used for association analysis where a kinship matrix was generated using genotypic data from the genome wide scan to control for relatedness of the study participants. The distribution of data values for Aß40, Aß42 and totalAß did not meet the assumption of normality and so a rank transformation was implemented in GenABEL to normalize the data distributions. Using the polygenic and mmscore functions available in GenABEL we tested the additive effect of the minor allele (T) for this SNP on the transformed plasma Aß phenotypes including covariates for age and sex in the model. Using the functions available in the related R package MetABEL (http://mga.bionet.nsc.ru/~yurii/ABEL/) we performed meta analysis for this SNP using the Vis and Korcula populations. The results of this analysis are shown in **Table S5**.

### Preparation of *IDE* v311 Construct

A 149bp fragment containing v311 was cloned into a luciferase pGL3 vector containing an SV40 promoter (Promega) using the Gateway cloning system (Invitrogen). AttB-flanked primers specific to a 149bp region surrounding v311 (forward 5′ GGGGACAAGTTTGTACAAAAAAGCAGGCT-TGGTCATTTTGGAGATGTGG 3′, reverse 5′ GGGGACCACTTTGTACAAGAAAGCTGGGT-TCACACAGCATTGTTTTCCA 3′) were used to amplify genomic DNA extracted from individuals known to be homozygous for v311 major or minor allele. PCR reactions were performed in a reaction mix containing 1xPCR buffer containing 1.5mM MgCl_2_ (QIAGEN), 1mM dNTPs (Promega), 0.2µM each primer, 2.5U HotStar Taq DNA polymerase and 20ng genomic DNA to a final volume of 25µl. Amplification conditions were as follows; 95°C for 5 minutes, followed by 35 cycles of 95°C for 30 seconds, 54°C for 1 minute, 72°C for 1 minute and finally an extension step of 72°C for 10 minutes. The resultant amplicons (major and minor allele) were extracted from an ethidium bromide-stained agarose gel using a QIAquick Spin kit (QIAGEN) and verified by sequencing (Mayo Clinic, Rochester). The attB-flanked fragments were integrated via bacterial recombination into a kanamycin-resistant pDONR 221 vector using the BP Clonase II system (Invitrogen) to produce an entry clone. Entry clones were transformed into Library efficiency DH5α chemically competent E.coli (Invitrogen) and grown on LB agar containing 50µg/ml kanamycin overnight at 37°C. Single colonies were picked for inoculation in liquid LB broth containing 50µg/ml kanamycin and incubated overnight in a shaking incubator at 37°C. Plasmids were extracted from the bacterial cells using a QIAprep spin kit (QIAGEN). Final expression clones were constructed by recombination of the entry clones with ampicillin-resistant pGL3 promoter vector using the LR Clonase II system (Invitrogen). Expression clones were transformed into DH5α E.Coli and grown on LB agar containing 100µg/ml ampicillin and single colonies were inoculated in LB broth containing 100µg/ml ampicillin. Plasmids were extracted using endotoxin-free Zyppy Plasmid miniprep kit (Zymo research) and verified by sequencing. Four expression clones were made in total; 5′G and 5′A contained 311 major (G) or minor (A) sequence positioned 5′ to the SV40 promoter and luciferase reporter gene, 3′G and 3′A contained sequence positioned 3′ to the luciferase gene.

### Cell Culture

Human HepG2 hepatocellular carcinoma and Be(2)-C neuroblastoma immortalized cell lines were supplied by ATCC. Cells were cultured in Eagle Minimum Essential Medium (EMEM) supplemented with 10% fetal bovine serum, 2mM L-Glutamine, 1× non-essential amino acids, 1000U/ml Penicillin-Streptomycin (Sigma), 2.5µg/ml Fungizone (Invitrogen). All cultures were incubated at 37°C in 5% CO_2_.

### Transfections

1×10^5^ Be(2)-C cells or 3×10^5^ HepG2 cells were plated in12-well culture plates 24 hours before transfection. Cells were co-transfected in triplicate with the v311 luciferase expression clones and a pRL vector (Promega) containing Renilla Luciferase reporter gene. Control wells included co-transfection of either pGL3 B (empty pGL3vector) or P (containing the SV40 promoter) with pRL. On day of transfection, cells were washed ×2 with PBS and media was replaced with 400µl serum-free EMEM containing 200ng expression clone or control vector, 10ng pRL and transfection reagent Tfx-20 (Promega) at a charge ratio of 3∶1 (Tfx∶DNA) per well. Transfection mix was pre-incubated for 15 minutes at room temperature. One hour after transfection, 800µl complete EMEM was added to each well.

### Dual Luciferase Assay

48 hours after transfection, cells were washed ×2 with PBS and harvested with 200µl of 1× Lysis buffer (Promega) for 20 minutes on a rocking platform. 5µl lysate was plated in a white 96-well assay plate. Firefly and Renilla luciferase signal were measured on a Veritas microplate luminometer (Turner Biosystems) using the Dual luciferase reporter assay system (Promega). The ratio of Firefly to Renilla luciferase signal was used to normalize firefly activity for intra-experimental transfection efficiency. Unpaired t-tests comparing mean relative firefly signal for our expression clones were performed using Stats Direct.

### Web Resources

dbSNP: http://www.ncbi.nlm.nih.gov/projects/SNP/References

Alzgene: http://www.alzforum.org/res/com/gen/alzgene/default.asp

## Supporting Information

Text S1 Discussion of single variant and haplotypic associations. Supplementary note.
(0.04 MB DOC)

Figure S1 Location and LD of common conserved *IDE* variants. (a) Location of the 68 *IDE* variants that meet conservation criteria (red vertical lines, conservation >70% identity over 100 bp bewteen human and mouse) is shown relative to *IDE* exons (blue boxes) and UTRs (green boxes). The gene structure is shown in the 5′ to 3′ orientation from p- to q-telomere. (b) Location of the 17 *IDE* variants that meet conservation and MAF criteria (MAF>1%). (c) HaploView LD D′ plot (solid spine block definition) and the 11 common haplotypes (frequency>1%) formed by these 17 variants.
(0.20 MB DOC)

Table S1 Details of samples used in this study. The number of AD patients (AD) and controls (CTRL), mean age, percentage that are female are given for each individual and pooled series. Mean age is given as age at diagnosis/entry. The standard deviation (SD) from the mean is given in parenthesis.
(0.07 MB DOC)

Table S2 Description of *IDE* conserved variants. Details for the 68 variants identified in our conserved variant screen are provided. The 17 common, conserved variants tested are in bold. Variant ID = “In house” unique variant identifier. rs = dbSNP variant identifier. Position = Chromosomal position based on the Human Genome build 36.1. Location = Variant location relative to the gene structure. a. a. Change = type of amino acid change predicted for variants within exons. Cons max = maximum conservation of any 100 bp window containing the variant. Study MAF = minor allele frequency in the combined USA+ART case control series. dbSNP MAF = minor allele frequency reported by dbSNP as of 10-17-08 (from HapMap-CEU, except for rs1042444 which only had CEPH MAF). dbSNP MAF: VNA = variant not present in dbSNP. dbSNP MAF: NA = MAF not reported in dbSNP. Source: a = found via dHPLC screen; b = found via SNPMiner; c = found via SNPMiner, but confirmed via dHPLC; d = found via dHPLC while confirming a different, neighboring variant found via SNPMiner.
(0.04 MB XLS)

Table S3 *IDE* variant information. The position of each variant is indicated relative to the Human Genome build 36.1. The columns labeled as 1 and 2 indicate the major and minor allele, respectively, and 11, 12, 22 indicate the corresponding genotype counts in all USA and ART combined series for the ten tagging variants (boldface type) and in the USA series for the remaining 7. MAF = minor allele frequency in Controls, Cons = conservation, rs = dbSNP variant identifier, HWp = Hardy-Weinberg p value in controls.
(0.10 MB DOC)

Table S4 *IDE* haplotypes. Haplotypes are numbered according to their frequency in the USA series. Only haplotypes with frequency >0.01 are shown. The “Haplotype” and “Variant ID” columns show the allelic composition of each haplotype in the 5′ to 3′ orientation from the p to the q telomere of chromosome 10. The allelic composition of each haplotype is depicted as “0” for major allele and “1” for minor allele. The 10 haplotype tagging variants are highlighted in bold.
(0.08 MB DOC)

Table S5 Illumina variant (rs7910977), which is in complete LD with v311, showing association with decreased plasma levels of Aβ40 and Aβ42. Association with plasma Aβ was performed using multivariable regression analysis providing coefficient values (negative coefficient represents decrease in plasma Aβ levels).
(0.05 MB DOC)
