# Supplementary material for: Concordant Association of Insulin Degrading Enzyme Gene (IDE) Variants with IDE mRNA, Aß, and Alzheimer's Disease
Source: PLoS One. 2010 Jan 19;5(1):e8764. doi: 10.1371/journal.pone.0008764 (PMC2808243; doi:10.1371/journal.pone.0008764)
Supplement: Text S1 — Discussion of single variant and haplotypic associations. Supplementary note. (0.04 MB DOC) [file pone.0008764.s001.doc]

##### Concordant association of insulin degrading enzyme gene (IDE) variants with IDE mRNA, Aß, and Alzheimer’s disease

##### Text S1: Discussion of single variant and haplotypic associations

Variant 311 (rs6583817) carriers constituted 18% of the samples in which *IDE* SNP/transcript association was analyzed, and linear regression showed that each additional copy of the minor allele of v311 was associated with a 2.12-fold higher (95% CI = 1.65-2.71) level of *IDE* transcript (p=1.5x10^-8^). Linkage disequilibrium is such (**Table S4**) that subjects who carry v311 can be divided into those who carry v311 with the v10-v315-v687 trio on haplotype 6 (11%), and those who carry v311 with v3 on haplotype 9 (7%). Haplotypes 6 (p=1.0x10^-5^) and 9 (p=0.006) both showed highly significant association with the level of *IDE* transcript (**Table 1**), with each additional copy of H6 being associated with 2.24-fold (95% CI = 1.56-3.21) higher levels of *IDE* transcript, and with each additional copy of H9 being associated with 2.34-fold (95% CI = 1.28-4.27) higher levels. The minor allele of v311 and the two haplotypes on which this allele is found were associated with essentially identical increases in the level of *IDE* transcript. This suggests a simple model in which v311 is postulated to be the only polymorphism that influences *IDE* function and risk for LOAD. In this simple model, the minor allele of v311 increases *IDE* expression slightly more than 2-fold, thereby reducing risk of LOAD.

However, additional associations that were observed raise questions about this simple model. For instance, H2 showed significant (p=0.03) association with a reduced level of *IDE* transcript. Similarly, H5 showed a significant (p=0.005) association with LOAD in the combined series, but this haplotype showed no association (p=0.49) with the level of *IDE* transcript (**Table 1**). Linear regression using an allelic dosage model indicated that the level of *IDE* transcript was reduced 0.79-fold (95% CI = 0.64-0.98) per copy of H2. This suggests that the minor alleles of v685 and/or v776, the pair that form the H2 haplotype, may reduce *IDE* mRNA thereby increasing risk for LOAD. Consistent with this, analysis of the combined case control series by univariate logistic regression using an additive model showed that H2 had an OR of 1.09 (0.98-1.21) with a p value of 0.13, indicating that H2 showed suggestive association with increased risk of LOAD. Finally, H6 and H9 were associated with a nearly identical elevation in cerebellar *IDE* transcript, but H9 showed significant (p=0.04) association with LOAD with an OR of 0.79 (0.63-0.99) whereas H6 showed only suggestive (p=0.11) association with LOAD with an OR of 0.89 (0.77-1.03). These observations suggest a more complicated model wherein H2, H5, H6, and H9 would be postulated to have independent effects. They suggest that H2 reduces *IDE* expression thereby increasing risk for LOAD. They suggest that H5, a haplotype that is not tagged well by any single variant, increases risk for LOAD through an unknown mechanism. Finally, they suggest that H9 may be more powerful than H6 in reducing risk for LOAD, even though the two haplotypes showed essentially identical association with elevated cerebellar *IDE* mRNA.

If a simple model is postulated where the minor allele of v311, which is found on H6 and H9, is the only allele that influences risk for LOAD, then H6 and H9 should have the same association with reduced risk of LOAD, and the other 9 haplotypes should have the same association with increased risk of LOAD relative to H6 and H9. In practice, chance alone will insure that H6 and H9 have different ORs and that the 9 putative risky haplotypes have differing ORs. Because v311 has a modest effect on risk for LOAD, it is likely that some of the 11 haplotypes (e.g. H5) will show significant association and that others will not. There should, however, be no significant difference between H6 and H9 or between any of the 9 remaining haplotypes postulated to increase risk equivalently. Using H6 as the reference haplotype, H6 and H9 were not significantly different when the eleven haplotypes in the combined series were analyzed by multivariate logistic regression adjusting for sex, age at diagnosis, *APOE* ε4 dosage, and series effects. Similar analyses of the 9 remaining haplotypes also showed no differences that would eliminate the simple model proposed. However, it is difficult to achieve adequate power to discriminate between effects such as those observed for H6 and H9. Even with the large sample size in our case-control study, there was only 19% power to detect the relative difference of 1.13 observed between the odds ratios for H6 and H9. Similarly, there was less than 80% power to observe a p value less than 0.05, and thereby conclude that the full collection of haplotypes provided a better explanation than v311 alone in discriminating between LOAD cases and controls. Thus our study has insufficient power to distinguish unequivocally between a simple model which postulates that v311 is the only *IDE* variant with any influence on risk for LOAD and more complicated model(s) that may better explain the full collection of observed results.
