# Supplementary material for: Concordant Association of Insulin Degrading Enzyme Gene (IDE) Variants with IDE mRNA, Aß, and Alzheimer's Disease
Source: PLoS One. 2010 Jan 19;5(1):e8764. doi: 10.1371/journal.pone.0008764 (PMC2808243; doi:10.1371/journal.pone.0008764)
Supplement: Figure S1 — Location and LD of common conserved IDE variants. (a) Location of the 68 IDE variants that meet conservation criteria (red vertical lines, conservation >70% identity over 100 bp bewteen human and mouse) is shown relative to IDE exons (blue boxes) and UTRs (green boxes). The gene structure is shown in the 5′ to 3′ orientation from p- to q-telomere. (b) Location of the 17 IDE variants that meet conservation and MAF criteria (MAF>1%). (c) HaploView LD D′ plot (solid spine block definition) and the 11 common haplotypes (frequency>1%) formed by these 17 variants. (0.20 MB DOC) [file pone.0008764.s002.doc]

# Concordant association of insulin degrading enzyme gene (*IDE*) variants with *IDE* mRNA, Aß, and Alzheimer’s disease

**Figure S1.** **Location and LD of common conserved *IDE* variants.** (**a**) Location of the 68 *IDE* variants that meet conservation criteria (red vertical lines, conservation > 70% identity over 100 bp bewteen human and mouse) is shown relative to *IDE* exons (blue boxes) and UTRs (green boxes). The gene structure is shown in the 5’to 3’ orientation from p- to q-telomere. (**b**) Location of the 17 *IDE* variants that meet conservation and MAF criteria (MAF > 1%). (**c**) HaploView LD D’ plot (solid spine block definition) and the 11 common haplotypes (frequency > 1%) formed by these 17 variants.
