# Supplementary material for: Concordant Association of Insulin Degrading Enzyme Gene (IDE) Variants with IDE mRNA, Aß, and Alzheimer's Disease
Source: PLoS One. 2010 Jan 19;5(1):e8764. doi: 10.1371/journal.pone.0008764 (PMC2808243; doi:10.1371/journal.pone.0008764)
Supplement: Table S1 — Details of samples used in this study. The number of AD patients (AD) and controls (CTRL), mean age, percentage that are female are given for each individual and pooled series. Mean age is given as age at diagnosis/entry. The standard deviation (SD) from the mean is given in parenthesis. (0.07 MB DOC) [file pone.0008764.s003.doc]

# Concordant association of insulin degrading enzyme gene (*IDE*) variants with *IDE* mRNA, Aß, and Alzheimer’s disease

**Table S1. Details of samples used in this study.** The number of AD patients (AD) and controls (CTRL), mean age, percentage that are female are given for each individual and pooled series. Mean age is given as age at diagnosis/entry. The standard deviation (SD) from the mean is given in parenthesis.

| Series | AD | CTRL | Total |  | Mean Age (SD) | |  | % Female | |
| --- | --- | --- | --- | --- | --- | --- | --- | --- | --- |
|  |  |  |  |  | AD | CTRL |  | AD | CTRL |
| USA |  |  |  |  |  |  |  |  |  |
| JS | 632 | 660 | 1,292 |  | 78.1 (6.1) | 77.5 (7.7) |  | 62.3 | 61.4 |
| RS | 576 | 1,417 | 1,993 |  | 79.7 (7.9) | 78.4 (6.0) |  | 63.4 | 54.6 |
| AUT | 603 | 374 | 977 |  | 81.1 (8.6) | 75.7 (8.3) |  | 59.2 | 42.8 |
| NCRAD | 702 | 209 | 911 |  | 75.2 (6.8) | 78.3 (8.9) |  | 64.8 | 61.7 |
| Total | 2,513 | 2,660 | 5,173 |  | 78.4 (7.7) | 77.8 (7.1) |  | 62.5 | 55.2 |
|  |  |  |  |  |  |  |  |  |  |
| ART |  |  |  |  |  |  |  |  |  |
| Belfast | 237 | 234 | 471 |  | 78.4 (6.7) | 76.2 (7.5) |  | 65.8 | 65.4 |
| Bonn | 175 | 199 | 374 |  | 75.9 (7.6) | 71.0 (6.9) |  | 76.0 | 49.2 |
| Manchester | 210 | 0 | 210 |  | 68.7 (6.3) | N/A |  | 50.0 | N/A |
| Oxford | 167 | 205 | 372 |  | 73.0 (6.8) | 77.3 (8.1) |  | 55.7 | 57.1 |
| Southampton | 227 | 143 | 370 |  | 80.9 (7.5) | 75.8 (6.6) |  | 59.0 | 49.0 |
| Total | 1,016 | 781 | 1,797 |  | 75.6 (8.2) | 75.1 (7.8) |  | 61.1 | 56.1 |
|  |  |  |  |  |  |  |  |  |  |
| USA and ART | 3,529 | 3,441 | 6,970 |  | 77.6 (7.9) | 77.2 (7.3) |  | 62.1 | 55.4 |
