# Supplementary material for: Concordant Association of Insulin Degrading Enzyme Gene (IDE) Variants with IDE mRNA, Aß, and Alzheimer's Disease
Source: PLoS One. 2010 Jan 19;5(1):e8764. doi: 10.1371/journal.pone.0008764 (PMC2808243; doi:10.1371/journal.pone.0008764)
Supplement: Table S3 — IDE variant information. The position of each variant is indicated relative to the Human Genome build 36.1. The columns labeled as 1 and 2 indicate the major and minor allele, respectively, and 11, 12, 22 indicate the corresponding genotype counts in all USA and ART combined series for the ten tagging variants (boldface type) and in the USA series for the remaining 7. MAF = minor allele frequency in Controls, Cons = conservation, rs = dbSNP variant identifier, HWp = Hardy-Weinberg p value in controls. (0.10 MB DOC) [file pone.0008764.s005.doc]

# Concordant association of insulin degrading enzyme gene (*IDE*) variants with *IDE* mRNA, Aß, and Alzheimer’s disease

**Table S3. *IDE* variant information.** The position of each variant is indicated relative to the Human Genome build 36.1. The columns labeled as 1 and 2 indicate the major and minor allele, respectively, and 11, 12, 22 indicate the corresponding genotype counts in all USA and ART combined series for the ten tagging variants (boldface type) and in the USA series for the remaining 7. MAF = minor allele frequency in Controls, Cons = conservation, rs = dbSNP variant identifier, HWp = Hardy-Weinberg p value in controls.

| ID | rs | Chr  position | Gene  Location | Cons. | MAF | 1 | 2 | AD | | |  | Control | | | HWp |
| --- | --- | --- | --- | --- | --- | --- | --- | --- | --- | --- | --- | --- | --- | --- | --- |
|  |  |  |  |  |  |  |  | 11 | 12 | 22 |  | 11 | 12 | 22 |  |
| **2** | N/A | 94,202,383 | 3' flank | 81% | 0.020 | AA | -- | 3,343 | 157 | 3 |  | 3,297 | 130 | 3 | 0.15 |
| **3** | rs5786996 | 94,202,516 | 3' flank | 74% | 0.041 | - | C | 3,287 | 228 | 5 |  | 3,142 | 277 | 1 | 0.04 |
| **6** | rs5786997 | 94,203,071 | 3' flank | 88% | 0.108 | -- | AT | 2,685 | 620 | 5 |  | 2,666 | 643 | 39 | 0.93 |
| 10 | rs4646958 | 94,204,339 | Intron 24 | 75% | 0.096 | A | T | 2,101 | 375 | 24 |  | 2,156 | 471 | 19 | 0.26 |
| **154** | rs4646957 | 94,219,892 | Intron 18 | 71% | 0.355 | G | A | 1,440 | 1,575 | 416 |  | 1,382 | 1,530 | 418 | 0.88 |
| 309 | N/A | 94,236,972 | Exon 13 | 92% | 0.017 | G | T | 2,344 | 117 | 3 |  | 2,538 | 89 | 1 | 0.55 |
| **310** | N/A | 94,237,153 | Intron 12 | 72% | 0.017 | T | C | 3,363 | 120 | 0 |  | 3,251 | 113 | 0 | 1.00 |
| **311** | rs6583817 | 94,237,227 | Intron 12 | 74% | 0.132 | G | A | 2,659 | 698 | 52 |  | 2,557 | 787 | 55 | 0.60 |
| **176** | rs17875327 | 94,264,789 | Intron 4 | 85% | 0.103 | T | C | 2,806 | 629 | 34 |  | 2,728 | 658 | 23 | 0.01 |
| 315 | rs7895832 | 94,266,506 | Intron 4 | 83% | 0.091 | T | A | 2,099 | 353 | 19 |  | 2,175 | 450 | 16 | 0.20 |
| **46** | rs4646955 | 94,284,271 | Intron 3 | 76% | 0.247 | T | C | 1,928 | 1,286 | 238 |  | 1,948 | 1,236 | 224 | 0.14 |
| **684** | rs17107721 | 94,288,480 | Intron 1 | 71% | 0.050 | G | A | 3,131 | 301 | 11 |  | 3,045 | 324 | 7 | 0.72 |
| 180 | rs11187060 | 94,294,112 | Intron 1 | 70% | 0.340 | G | A | 1,116 | 1,095 | 256 |  | 1,128 | 1,221 | 286 | 0.11 |
| 683 | rs17445328 | 94,295,169 | Intron 1 | 72% | 0.250 | A | G | 1,368 | 913 | 164 |  | 1,439 | 953 | 163 | 0.75 |
| **685** | rs11187061 | 94,295,389 | Intron 1 | 70% | 0.184 | C | T | 2,268 | 1,059 | 141 |  | 2,263 | 1,009 | 120 | 0.57 |
| 687 | rs17107734 | 94,295,397 | Intron 1 | 70% | 0.091 | C | T | 2,086 | 358 | 19 |  | 2,160 | 445 | 16 | 0.20 |
| 776 | rs11187074 | 94,316,926 | Intron 1 | 73% | 0.181 | C | G | 1,594 | 725 | 99 |  | 1,714 | 748 | 86 | 0.69 |
