# Supplementary material for: Concordant Association of Insulin Degrading Enzyme Gene (IDE) Variants with IDE mRNA, Aß, and Alzheimer's Disease
Source: PLoS One. 2010 Jan 19;5(1):e8764. doi: 10.1371/journal.pone.0008764 (PMC2808243; doi:10.1371/journal.pone.0008764)
Supplement: Table S4 — IDE haplotypes. Haplotypes are numbered according to their frequency in the USA series. Only haplotypes with frequency >0.01 are shown. The “Haplotype” and “Variant ID” columns show the allelic composition of each haplotype in the 5′ to 3′ orientation from the p to the q telomere of chromosome 10. The allelic composition of each haplotype is depicted as “0” for major allele and “1” for minor allele. The 10 haplotype tagging variants are highlighted in bold. (0.08 MB DOC) [file pone.0008764.s006.doc]

# Concordant association of insulin degrading enzyme gene (*IDE*) variants with *IDE* mRNA, Aß, and Alzheimer’s disease

**Table S4. *IDE* haplotypes.** Haplotypes are numbered according to their frequency in the USA series. Only haplotypes with frequency > 0.01 are shown. The "Haplotype" and "Variant ID" columns show the allelic composition of each haplotype in the 5' to 3' orientation from the p to the q telomere of chromosome 10. The allelic composition of each haplotype is depicted as "0" for major allele and "1" for minor allele. The 10 haplotype tagging variants are highlighted in bold.

| Haplotype | Freq | Variant ID | | | | | | | | | | | | | | | | |
| --- | --- | --- | --- | --- | --- | --- | --- | --- | --- | --- | --- | --- | --- | --- | --- | --- | --- | --- |
|  |  | 2 | 3 | 6 | 10 | 154 | 309 | 310 | 311 | 176 | 315 | 46 | 684 | 180 | 683 | 685 | 687 | 776 |
| H1 | 0.23 | 0 | 0 | 0 | 0 | 0 | 0 | 0 | 0 | 0 | 0 | 0 | 0 | 0 | 0 | 0 | 0 | 0 |
| H2 | 0.18 | 0 | 0 | 0 | 0 | 0 | 0 | 0 | 0 | 0 | 0 | 0 | 0 | 0 | 0 | **1** | 0 | 1 |
| H3 | 0.10 | 0 | 0 | 0 | 0 | 1 | 0 | 0 | 0 | **1** | 0 | 1 | 0 | 1 | 1 | 0 | 0 | 0 |
| H4 | 0.10 | 0 | 0 | **1** | 0 | 0 | 0 | 0 | 0 | 0 | 0 | 0 | 0 | 0 | 0 | 0 | 0 | 0 |
| H5 | 0.10 | 0 | 0 | 0 | 0 | **1** | 0 | 0 | 0 | 0 | 0 | 1 | 0 | 1 | 1 | 0 | 0 | 0 |
| H6 | 0.08 | 0 | 0 | 0 | 1 | 1 | 0 | 0 | **1** | 0 | 1 | 0 | 0 | 1 | 0 | 0 | 1 | 0 |
| H7 | 0.05 | 0 | 0 | 0 | 0 | 0 | 0 | 0 | 0 | 0 | 0 | **1** | 0 | 0 | 1 | 0 | 0 | 0 |
| H8 | 0.05 | 0 | 0 | 0 | 0 | 0 | 0 | 0 | 0 | 0 | 0 | 0 | **1** | 0 | 0 | 0 | 0 | 0 |
| H9 | 0.03 | 0 | **1** | 0 | 0 | 1 | 0 | 0 | 1 | 0 | 0 | 0 | 0 | 1 | 0 | 0 | 0 | 0 |
| H10 | 0.02 | **1** | 0 | 0 | 0 | 1 | 1 | 0 | 0 | 0 | 0 | 0 | 0 | 0 | 0 | 0 | 0 | 0 |
| H11 | 0.02 | 0 | 0 | 0 | 0 | 0 | 0 | **1** | 0 | 0 | 0 | 0 | 0 | 0 | 0 | 0 | 0 | 0 |
