# Supplementary material for: Concordant Association of Insulin Degrading Enzyme Gene (IDE) Variants with IDE mRNA, Aß, and Alzheimer's Disease
Source: PLoS One. 2010 Jan 19;5(1):e8764. doi: 10.1371/journal.pone.0008764 (PMC2808243; doi:10.1371/journal.pone.0008764)
Supplement: Table S5 — Illumina variant (rs7910977), which is in complete LD with v311, showing association with decreased plasma levels of Aβ40 and Aβ42. Association with plasma Aβ was performed using multivariable regression analysis providing coefficient values (negative coefficient represents decrease in plasma Aβ levels). (0.05 MB DOC) [file pone.0008764.s007.doc]

# Concordant association of insulin degrading enzyme gene (*IDE*) variants with *IDE* mRNA, Aß, and Alzheimer’s disease

**Table S5.** **Illumina variant (rs7910977), which is in complete LD with v311, showing association with decreased plasma levels of Aβ40 and Aβ42.** Association with plasma Aβ was performed using multivariable regression analysis providing coefficient values (negative coefficient represents decrease in plasma Aβ levels).

| rs number | Chr | Study population | Aβ40 | | |  | Aβ42 | | |  | Total Aβ | | |
| --- | --- | --- | --- | --- | --- | --- | --- | --- | --- | --- | --- | --- | --- |
| (minor allele) | position |  | beta | SE | p |  | beta | SE | p |  | beta | SE | p |
| rs7910977 (T) | 94199856 | Vis | -0.147 | 0.06 | **0.018** |  | -0.103 | 0.07 | 0.13 |  | -0.165 | 0.06 | **0.011** |
|  |  | Korcula | -0.086 | 0.08 | 0.27 |  | -0.024 | 0.08 | 0.76 |  | -0.080 | 0.08 | 0.31 |
|  |  | Meta Analysis | -0.124 | 0.05 | **0.011** |  | -0.07 | 0.05 | 0.18 |  | -0.130 | 0.05 | **0.009** |
